# Novel quantification of extracellular expansion by cardiac magnetic resonance is a robust marker in diagnosis of cardiac amyloidosis

**DOI:** 10.1186/1532-429X-13-S1-P301

**Published:** 2011-02-02

**Authors:** Francois-Pierre Mongeon, Michael Jerosch-Herold, Otavio Rizzi Coelho-Filho, Luciana F Seabra, Eri Watanabe, Ron Blankstein, Raymond Y Kwong

**Affiliations:** 1 Brigham and Women's Hospital, Boston, MA, USA

## Background

Precise quantification of myocardial amyloid burden may improve the diagnosis and monitoring of cardiac amyloidosis (CA). While late gadolinium enhancement (LGE) imaging by MRI demonstrates a characteristic pattern of infiltration, it does not quantify the extent of myocardial extracellular matrix expansion which occurs in CA. We tested the hypothesis that a direct measure of myocardial extracellular volume fraction (MECVF) using T1-weighted imaging pre- and post-contrast can identify CA.

## Methods

We performed 3T cardiac MRI in 47 subjects including 9 control subjects (mean age 45±11 years, 66.7% female) and 38 consecutive patients (mean age 68±15 years, 23.7% female) referred with (1) known CA, (2) unexplained left ventricular hypertrophy or (3) a systemic disease susceptible of infiltrating the myocardium (2 amyloidosis, 2 monoclonal gammopathy). MRI included cine imaging and LGE 10 min after 0.15 mmol/Kg of gadolinium. In addition, a Look-Locker gradient echo technique with adiabatic inversion was used, once before and 3 times over a 30-min period after gadolinium injection, to quantify T1. The myocardial partition coefficient was estimated by least-squares linear regression of R1 (1/T1) in myocardium against R1 in blood. MECVF was obtained by adjusting the partition coefficient by the patient’s hematocrit. LV subendocardial and subepicardial LGE was blindly graded as present or absent using a 17-segment model.

## Results

**Figure 1:**
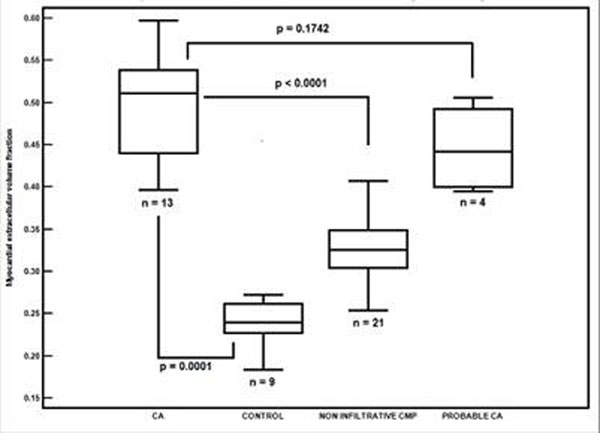
Myocardial extracellular volume fraction according to final diagnosis

In this cohort, 13 (27.6%) patients were confirmed to have CA by biopsy and 4 (8.5%) were diagnosed with probable CA by clinical evaluation, 21 (44.7%) patients had a non infiltrative cardiomyopathy (7 hypertensive cardiomyopathies, 2 patients with ventricular arrhythmias, 1 with an extracardiac glycogen storage disease, 1 hypertrophic cardiomyopathy, 10 cardiomyopathies of undefined etiology). Mean MECVF was substantially higher in patients with CA than in the non infiltrative cardiomyopathy group and in normal controls (p<0.0001 vs. non infiltrative cardiomyopathy, p=0.0001 vs. controls and p=0.1742 vs. probable CA, Figure 1). MECVF demonstrated a strong correlation with the extent of LGE (r=0.77, p<0.0001) and with LV mass index (r=0.69, p<0.0001) and an inverse correlation with LVEF (r=-0.55, p=0.0002).

## Conclusions

Direct quantification of extracellular matrix expansion by MECVF may characterize the burden of CA infiltration. Elevated MECVF is associated with higher extent of LGE and increased LV mass index. This novel quantitative method may aid in the differential diagnosis of infiltrative heart disease and in the recognition of CA. It also holds promises for monitoring the response to therapy.

